# Soilless Plant Growth Media Influence the Efficacy of Phytohormones and Phytohormone Inhibitors

**DOI:** 10.1371/journal.pone.0107689

**Published:** 2014-12-08

**Authors:** Norman B. Best, Thomas Hartwig, Joshua S. Budka, Brandon J. Bishop, Elliot Brown, Devi P. V. Potluri, Bruce R. Cooper, Gnanasiri S. Premachandra, Cliff T. Johnston, Burkhard Schulz

**Affiliations:** 1 Department of Horticulture and Landscape Architecture, Purdue University, West Lafayette, Indiana, United States of America; 2 Department of Plant Biology, Carnegie Institution for Science, Stanford, California, United States of America; 3 Plant Soil and Nutrition Research Unit, USDA ARS, Ithaca, New York, United States of America; 4 Department of Biology, Chicago State University, Chicago, Illinois, United States of America; 5 Bindley Bioscience Center, Purdue University, West Lafayette, Indiana, United States of America; 6 Department of Agronomy, Purdue University, West Lafayette, Indiana, United States of America; University of Nottingham, United Kingdom

## Abstract

Plant growth regulators, such as hormones and their respective biosynthesis inhibitors, are effective tools to elucidate the physiological function of phytohormones in plants. A problem of chemical treatments, however, is the potential for interaction of the active compound with the growth media substrate. We studied the interaction and efficacy of propiconazole, a potent and specific inhibitor of brassinosteroid biosynthesis, with common soilless greenhouse growth media for rice, sorghum, and maize. Many of the tested growth media interacted with propiconazole reducing its efficacy up to a hundred fold. To determine the molecular interaction of inhibitors with media substrates, Fourier Transform Infrared Spectroscopy and sorption isotherm analysis was applied. While mica clay substrates absorbed up to 1.3 mg of propiconazole per g substrate, calcined clays bound up to 12 mg of propiconazole per g substrate. The efficacy of the gibberellic acid biosynthesis inhibitor, uniconazole, and the most active brassinosteroid, brassinolide, was impacted similarly by the respective substrates. Conversely, gibberellic acid showed no distinct growth response in different media. Our results suggest that the reduction in efficacy of propiconazole, uniconazole, and brassinolide in bioassays when grown in calcined clay is caused by hydrophobic interactions between the plant growth regulators and the growth media. This was further confirmed by experiments using methanol-water solvent mixes with higher hydrophobicity values, which reduce the interaction of propiconazole and calcined clay.

## Introduction

**Figure 1:**
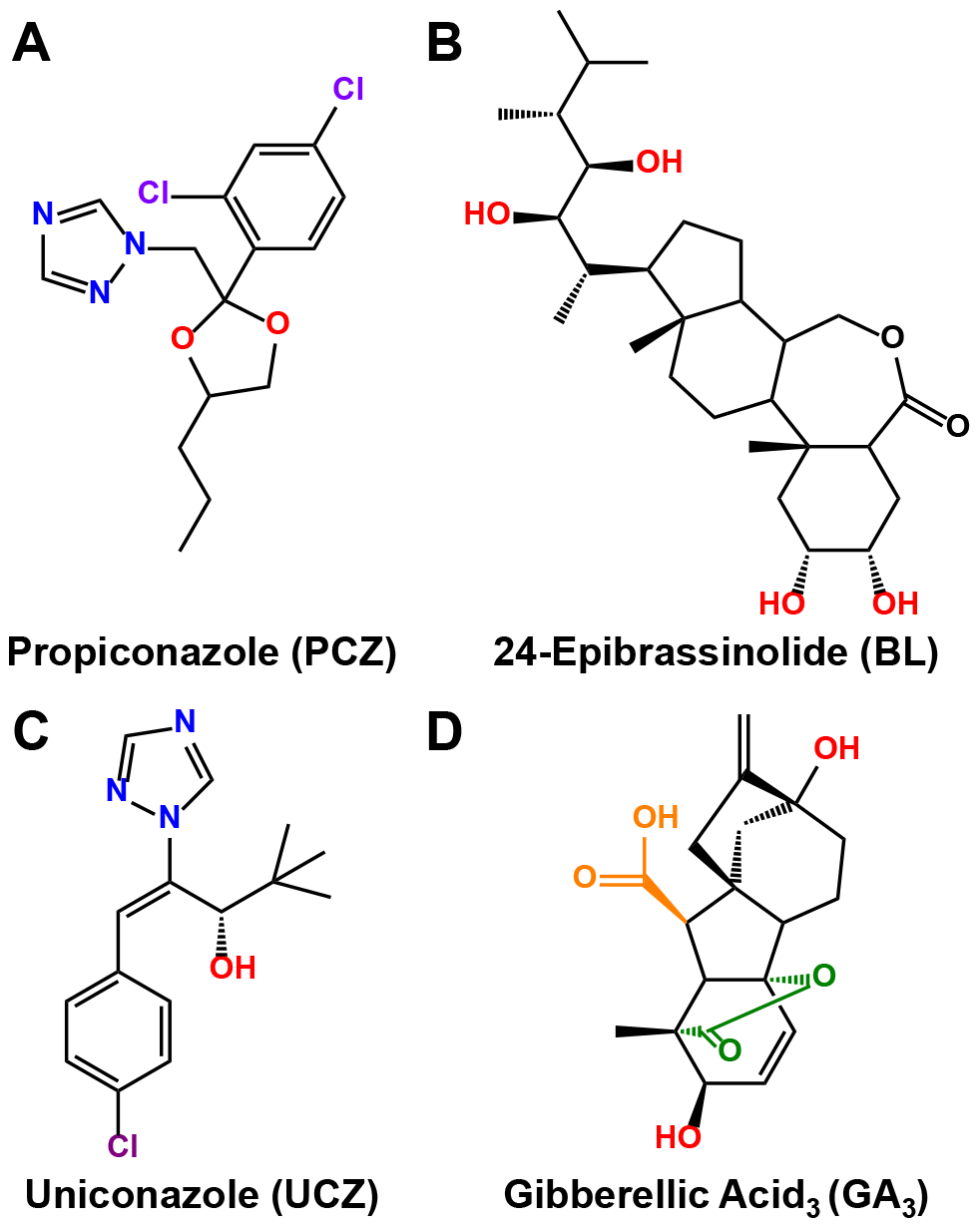
Chemical structures of propiconazole (Pcz), uniconazole (Ucz). 24-epibrassinolide (eBL), and gibberellic acid_3_ (GA_3_). Structure elements critical for inhibitor activity in Pcz and Ucz have been color-coded: (blue) nitrogen atoms in the azole ring; (purple) chlorine atom(s) of the phenyl ring; and (red) either primary/secondary hydroxyl group or 1,3-dioxlane. In GA_3_, the 4,10-lactone bridge is depicted in green and the carboxyl group is depicted in orange. Hydroxyl groups in 24-epibrassinolide and GA_3_ are depicted in red. Structures were drawn using the ChemBioDraw 12.0.2 software and structures were compared to the ChemACX 12.12.1 database.

Biochemical treatments, in addition to mutant studies, are very effective approaches to study the function of endogenous signal substances such as phytohormones. Our understanding of both the biosynthetic as well as signaling pathways of plant hormones, such as gibberellic acid (GA) and brassinosteroids (BRs), benefited greatly from the use of chemical inhibitors [1]–[2]. GA is a tetracyclic dihydroxy lactonic acid first identified from culture filtrates of the fungus *Gibberella fujikuroi*, whereas BRs were first identified in pollen of *Brassica napus* and are polyhydroxylated steroidal hormones (Fig. 1) [3]–[4]. Both GAs and BRs are major plant growth regulators (PGRs) and show substantial overlap in the developmental processes that they affect. Mutants deficient in either BRs or GAs exhibit dwarfism in skoto- and photomorphogenesis, reduced seed germination, and delayed flowering [5]–[7]. On the other hand, GAs and BRs can also have opposite functions as exhibited by their effects on sex determination in maize [8]–[9].

A number of triazole compounds have been shown to inhibit cytochrome P450 enzymes, which are key components of both, GA as well as BR biosynthesis. Uniconazole (Ucz) and brassinazole (Brz) are two of the classical inhibitors shown to interact with CYP701 of the GA and CYP90B1 of the BR biosynthetic pathways, respectively. Although well characterized in the model plant *Arabidopsis thaliana*, comprehensive understanding of the biological function of BRs in agronomical crops such as maize is still lacking. One of the limitations that have hindered research progress is availability and cost of some of the PGRs. While Ucz and GA are easily accessible and found use in horticultural greenhouse production [10]–[12]; cost and availability of both Brz and brassinolide (BL) greatly restrict their use to study BR function in agronomic species [13]. Propiconazole (Pcz), 1-[[2-(2,4-dichlorophenyl)-4-propyl-1,3-dioxolan-2-yl]methyl]-1,2,4-triazole, was recently characterized in *Arabidopsis* and maize as an alternative BR inhibitor (Fig. 1). Not only does Pcz act as a potent and more specific BR inhibitor than Brz, but better accessibility and drastically reduced cost alleviates the major limitations of Brz for the use with larger plants [1], [13]. Pcz is a major fungicide for treatment of dollar spot disease (*Sclerotinia homoeocarpa*) in turf grasses on golf courses and sod farms. It is also registered for use as fungicide in maize. Global sales of Pcz exceeded US$ 200 million in 1995 [14] and its use is predicted to rise due to increased global sales for fungicides in the coming years. The fungicidal effect of Pcz is based on its ability to suppress the biosynthesis of ergosterol, which is an essential sterol component of the fungal cell membrane. Pcz binds to the heme group of 14α-demethylase cytochrome P450 enzymes via its azole ring, which results in inactivation of the ergosterol biosynthesis in fungi [15] and subsequent cell death. Cytochrome P450 enzymes are also involved in important steps of the BR biosynthesis in plants [16], [17]. Asami *et al*. [18] showed that triazole derivatives with BR inhibitor activity, related to Pcz, are able to bind and inhibit the rate-limiting step of the BR biosynthesis, the steroid 22-hydroxylase cytochrome P450 enzyme DWF4.

Plant growth experiments often require the use of various growth media substrates to control for water retaining potential, nutrient availability, and anchoring of roots in the media. Murashige and Skoog (MS) agar-based media was developed for tobacco as a suitable substrate that allows rapid growth and shows minimal side effects on bioassays and chemical treatments in tissue culture [19]. Although MS is useful for seedling experiments on smaller plants such as *Arabidopsis*, growing mature crops in MS is often difficult and cost inefficient. To achieve optimal growth under greenhouse conditions, larger crops such as maize, rice, or soybean often require a variety of soilless growth substrates varying from sphagnum peat moss to calcined clay. Furthermore, perlite or vermiculite additives are often used to improve substrate aeration and soil health to increase growth and yield. Whereas agar-based MS media was designed to be neutral in its interactions with chemicals, most of the soilless growth substrates have not been studied for their potential to influence the efficacy of biochemical treatments. Previous work showed that Pcz in different soils is not very mobile and had a high retention time [20]–[22].

The interaction of PGR with growth media has been an important research topic for the ornamental plant industry for many years [23], [24], [10]. Triazole compounds commonly used in ornamental crop production such as Ucz and paclobutrazol (Pac) have been tested on a wide array of species, mostly in peat-based substrates [25]–[36]. Additives to soilless peat media, such as pine bark, vermiculite, or rice hulls were also tested for their effects on PGR efficacy in multiple studies [37]–[41]. Despite numerous investigations in this area, very little is known about how these components interact with PGRs. Particularly relevant to our study is the interaction with calcined clays, which provide optimal growth conditions for container culture of larger crop species such as maize.

Here, we present the characterization of the four most commonly used greenhouse growth substrates vermiculite, perlite, sphagnum peat moss, calcined clay, or combinations thereof, and their interactions with Pcz, 24-epibrassinolide (eBL), Ucz, or GA_3_. The efficacy of Pcz varied by more than 100-fold between media substrates tested. The ability of calcined clay substrates to interfere with the efficacy of triazole-based PGRs is based on the hydrophobic interactions of PGRs with the substrates. Our data suggests that various media substrates have a significant impact on the efficacy of chemical treatments and the reproducibility of such experiments, which illustrates the importance of appropriate media selection for the given experimental design. We show how differences in interaction of chemical compounds with growth substrates can be explained based on physicochemical properties of both interaction partners.

## Material and Methods

### SEM

Samples of various growth media substrates were mounted on aluminum stubs using a carbon tape adhesive. Samples were viewed using a JEOL 6610 LV Scanning Electron Microscope (Jeol, Peabody, MA) in standard vacuum mode at 5 kV accelerating voltage. The vermiculate has a layered structure so care was taken to view the surface from different angles. Initial attempts to view the surface of Turface resulted in substantial charging artifacts. To overcome this, Turface was sputter-coated with gold. This procedure resolved the charging issue.

### Growth of seedlings in soilless growth media and chemical treatments

Maize B73 seedlings were grown under greenhouse conditions at 27°C (day) and 21°C (night) with 16 h of supplemental lighting. Plants were fertilized with 200 ppm Miracle-Gro Excel (Scotts, Marysville, OH) adjusted to pH 6 following manufacturer recommendations. For all treatment experiments seeds were sterilized for 7 min at 60°C in a water bath prior to planting and grown under greenhouse conditions. Seeds were planted 5 cm deep in 24.5 cm wide pots with vermiculite extra-coarse grade (SunGro Horticulture, Bellevue, WA and Perlite Vermiculite Packaging Industries, Inc., North Bloomfield, OH), Turface MVP (Profile Products LLC, Buffalo Grove, IL), Perlite coarse grade (SunGro Horticulture, Bellevue, WA and Perlite Vermiculite Packaging Industries, Inc., North Bloomfield, OH), Peat germinating mix (Conrad Fafard Inc., Agawam, MA), or mixtures thereof and watered every fifth day. Pcz (Banner Maxx, Syngenta, Greensboro, NC) was added at indicated concentrations to the watering solution. After 21 d plants were harvested, photographed, and analyzed using ImageJ [42]. Plant height was measured from the root-shoot transition zone to the highest leaf collar.

For treatment experiments grown on Turface or vermiculite comparing Pcz, Ucz (Sumagic, Syngenta, Greensboro, NC), eBL (Sigma Aldrich, St Louis, MO) dissolved in 0.02% ethanol, or GA_3_ (Gold Biotechnology, St. Louis, MO) dissolved in 0.02% ethanol, B73 seedlings were grown under greenhouse conditions as previously stated. Seeds were planted 5 cm deep in 606 trays with respective growth media and treatment. After 9 d seedlings were harvested, photographed, and analyzed using ImageJ [42]. Plant height was measured from the root-shoot transition zone to the highest leaf collar using photographed length standards.

### HPLC-MS

HPLC-MS analysis was performed using an Agilent 1100 HPLC system (Agilent, Palo Alto, CA) with a Shimadzu Shim-pack XR-ODS column (75 × 3.0 mm). Isocratic elution was used, with a mobile phase composition of 40% deionized water and 60% acetonitrile, with a buffer of 0.1% formic acid (v/v). Mobile phase flow rate was 0.6 ml/min. Following separation the column effluent was introduced by positive mode electrospray ionization (ESI) into an Agilent MSD time-of-flight mass spectrometer. ESI capillary voltage was 3.5 kV. Nebulizer gas pressure was set at 55 psig, gas temperature was 350°C, and drying gas flow rate was 11 l/min. The fragmentor voltage was set to 135 V, skimmer 65 V and OCT RF V 250 V. Spectroscopic (UV 200 - 350 nm) and mass (m/z 70–1500) data were collected and analyzed using Agilent MassHunter software.

### FTIR

Turface was combined with 15.4 mg of Pcz in a 50 mL Falcon tube for 500 h to obtain Pcz+ Turface and was dried for 48 h in a drying oven at 50°C. FTIR analysis of Turface and Pcz+Turface was performed using a Thermo Scientific Smart Diffuse Reflectance accessory on a Thermo Scientific Nicole 6700 FT-IR spectrometer (Thermo Scientific, Waltham, MA). A mixture of 5% Turface or Pcz+Turface in KBr was prepared using oven dried KBr powder (10 mg Turface or Pcz+Turface and 190 mg of ground KBr) were weighed and placed in an agate vial and mized using a Wig-L-Bug for 30 seconds. The prepared mixture was then placed in a sample cup in the diffuse reflectance accessory. FTIR spectra were collected using a medium-band MCT detector in the spectral range of 4000 to 700 cm^−1^ region. A background spectrum was obtained using KBr (only) in the sample cup. FTIR spectra of Pcz and pure Pcz (95%) (ORico Orient Resources International Co. Ltd., Zhuhai, China) were obtained using a Pike GladiATR (Pike Technologies, Madison, WI) on a Thermo Scientific Nicolet 6700 FT-IR spectrometer (Thermo Scientific, Waltham, MA). A background spectrum was obtained from the clean ATR crystal. FTIR spectrum analysis was collected using OMNIC series software (Thermo Scientific, Waltham, MA).

### Adsorption isotherms of Turface and vermiculite

1 g of respective media was allowed to interact with 15.4 mg/40 ml of Pcz in a 50 mL Falcon tube for up to 265 h at room temperature in water or MeOH solvent mixes. Tubes were agitated on a rocker at 100 rpm. At each time point, a 15 µl aliquot of the supernatant was removed and centrifuged to remove excess media particles. Absorption was measured using a NanoDrop photometer (Thermo Scientific, Waltham, MA) at 225 nm. The isotherms were then calculated to determine the amount of Pcz adsorbed to the respective substrate over time.

### Aeroponics

Seeds were germinated in silica white sand (U.S. Silica, Frederick, MD) with the respective chemical treatment for 6 d. Seedlings were removed from growing media and sand was washed from roots, and seedlings were transferred to aeroponics cultivation device (S3A Figure). In aeroponic culture, the roots were finely misted with ¾ x modified Hoagland's solution [43], with double iron concentration, and the added respective chemical treatment for 3 s every 10 min. Seedlings were grown under greenhouse conditions with 14h supplemental lighting at 27°C (day) and 21°C (night). The root zone was cooled to 15°C with an AC block. Plants were harvested after 12 d (18 d total) of aeroponic culture, photographed, and analyzed using ImageJ software [42]. Plant height was measured from root-to-shoot transition zone to the second leaf collar and root length was measured from root-to-shoot transition zone to the tip of the longest root.

### X-ray diffraction analysis

X-ray diffraction patterns of Turface samples were obtained from oriented samples on glass slides [44] using a PANalytical X-ray diffractometer (Model X′Pert PRO; Natick, MA) using Co radiation. Data were from 2 to 80° 2θ, counting for 1s every 0.02° 2θ with a total scan time of 30.5 min. Data analysis was performed using X′Pert HighScore Plus software (Version 2.2, PANalytical B.V.).

### Statistics analysis

The Microsoft Excel add-in XL Toolbox (ver. 6.50, http://xltoolbox.sourceforge.net) was used to obtain all descriptive and comparative statistics. Analyses of variance (ANOVA) for sets of data groups were performed with “Multiple comparisons/Post-hoc” testing. Once a significant difference (*p*<0.05) was detected “Post-hoc” tests, using the Holm-Sidak algorithm, were performed to test for significance of the possible multiple comparisons between the data groups [45].

## Results

### Variance in growth media surface structure

**Figure 2:**
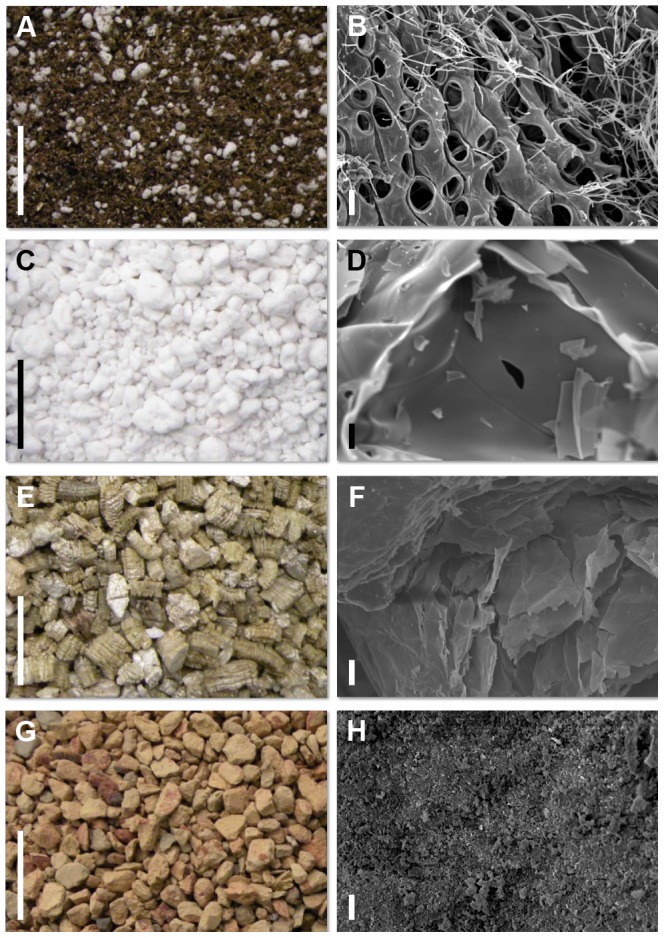
Photographic and SEM images of various media substrates. (A–B) peat germinating mix, (C–D) perlite. (E–F) vermiculite. (G–H) Turface. (A,C,E,G) Photographic images exhibiting textural differences between media substrates. (B,D,F,H) Scanning election microscopy photos of substrates. Scale Bar (A,C,E,H) 1 cm and (B,D,F,H) 10 µm.

The four media substrates chosen for this study not only differ in their chemical composition and water holding potential but also in their surface charge and affinity for water. Dried and decayed sphagnum peat moss is often used as a soil conditioner or growth substrate. Peat moss exhibits high water retention capacity compared to other common plant growth substrates due to its high capillary forces and cation exchange capacity (Fig. 2 A-B) [46]. Water retention curves (θ(ψ)) demonstrate that peat moss has one of the highest moisture retention ability at different moisture tension values [47]. Perlite on the other hand, is an amorphous volcanic glass that is produced through thermal expansion of hydrated obsidian and is primarily used as soil amendment or as hydroponic medium (Fig. 2 C–D). It has a high permeability and helps prevent soil compaction but suffers from low water retention [48]. Vermiculite is another thermal expanded soilless medium, which is produced by exfoliation of the silicate mineral mica (Fig. 2 E–F). It is structured in two tetrahedral sheets for every one octahedral sheet (2∶1) giving it a very high cation exchange capacity (CEC, 100–150 meq/100 g) and moderate water retention capacity [47]. Turface is a calcined, non-swelling illite and silica clay generated by thermal decomposition at around 650°C (Fig. 2 G–H). Despite its low water holding capacity, it is a preferred growth media for larger crop species such as maize due to its high binding and slow release properties for nutrients [49].

### Media substrates affect Pcz-induced growth reduction

**Figure 3:**
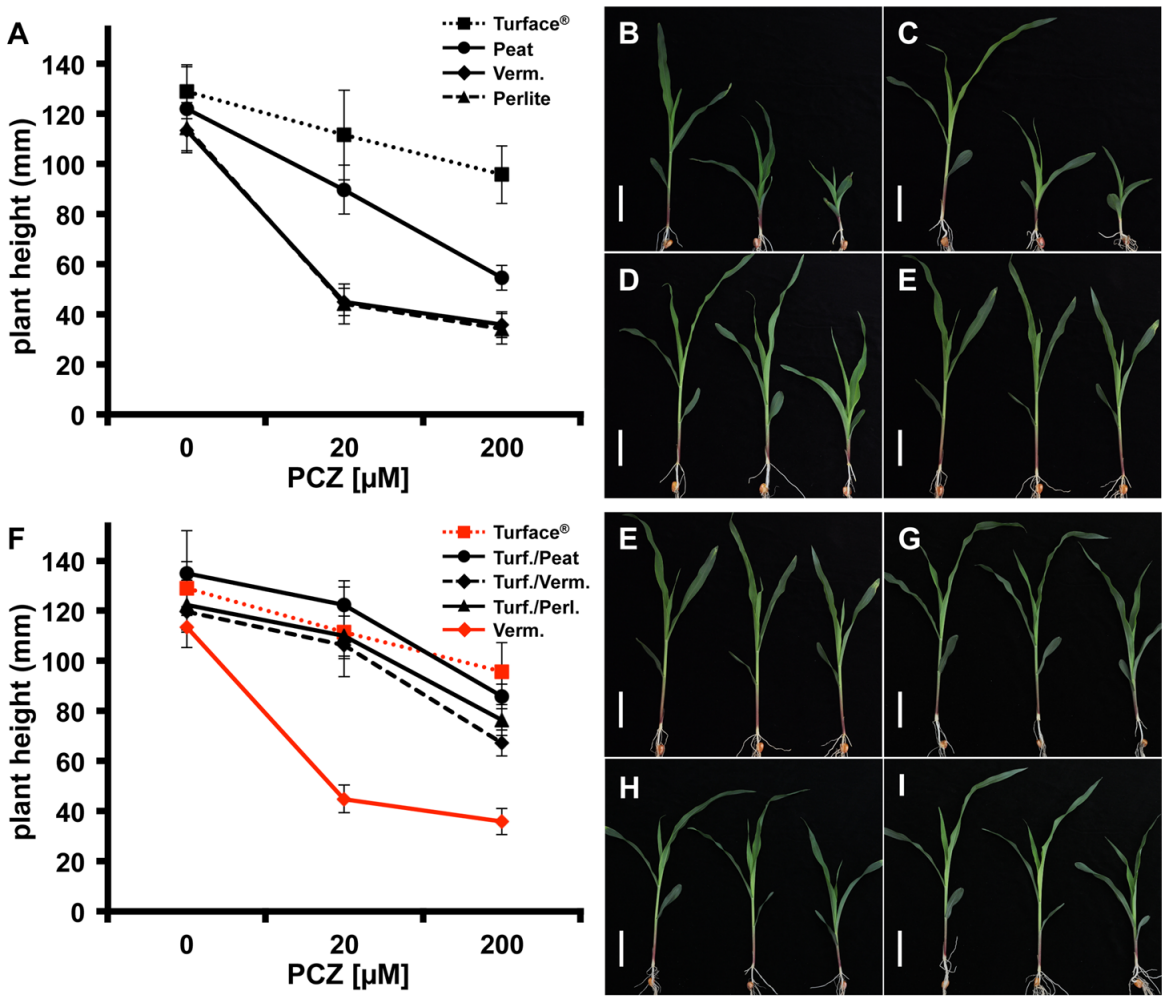
Plant growth response of maize seedlings treated with Pcz in different media substrates. (A–E) B73 maize seedlings grown for 11d in the light in either (B) Turface, (C) peat germinating mix, (D) vermiculite, or (E) perlite. (F–I) 1∶1 (v/v) mixtures of (G) Turface/peat mix, (H) Turface/vermiculite, and (I) Turface/perlite. Plants were watered without (left), 20 µM (center) or 200 µM (right) of Pcz as needed. (A) Height of plants grown in pure media as well as (F) Turface 1∶1 mixtures was measured from the root-shoot transition zone to the highest leaf collar using ImageJ 1.43. (A, F) Error bars represent standard deviation. Size bars indicate 5 cm.

Previous research indicated that Pcz is a potent and specific BR biosynthesis inhibitor and phenocopies BR deficient mutants in *Arabidopsis*, cress, and maize [1], [13]. One of the most striking phenotypes of maize seedlings treated with Pcz is their reduced growth resulting in a dwarf stature [9]. Dependent on the growth media used, we observed significant differences in the severity of Pcz-induced dwarf phenotypes. Pcz's ability to reduce plant growth was most effective when plants were grown in vermiculite or perlite. This effect was significantly lower in seedlings grown in peat moss and even more reduced in Pcz treated plants grown in Turface (Fig. 3 A–E). Seedlings of maize inbred line B73 grown in the presence of 20 µM Pcz in vermiculite were 60.5% shorter, compared to control seedlings (Fig. 3 A–B). Grown in perlite and treated with the same concentration of Pcz, B73 seedlings showed a similar height reduction of 61.5% (Fig. 3 A, 3C). On the other hand, 20 µM Pcz treatments produced only 26.5% shorter B73 seedlings when grown in peat moss mixes. Even more noticeable was the decreased effect of Pcz treatment on seedlings grown in Turface, which showed a mere 13.5% shorter stature compared to controls (Fig. 3 A, 3E). These results suggest that the tested media substrates significantly impact the efficacy of Pcz applied by soil drench treatments.

To further evaluate the impact of the growth media on Pcz efficacy we tested mixtures with equal parts (v/v) of Turface and the other three growth substrates, respectively. At a concentration of 20 µM, Pcz-mediated inhibition of plant height was not significantly different between plants grown in Turface and Turface 1∶1 mixtures (Fig. 3 F–I). Turface mixed equally with peat, perlite, or vermiculite resulted in height reductions of 9.4%, 10.1%, and 11%, respectively, compared to 13.5% shorter plants grown in pure Turface (Fig. 3 F–I). Pcz treatments at a concentration of 200 µM Pcz resulted in height reduction of 25.7% of plants grown in pure Turface, whereas 1∶1 mixtures of Turface with peat, perlite, or vermiculite caused 36.7%, 37.5%, or 43.7% height reduction, respectively (Fig. 3 F–I).

### Turface directly affects Pcz availability in solution

**Figure 4:**
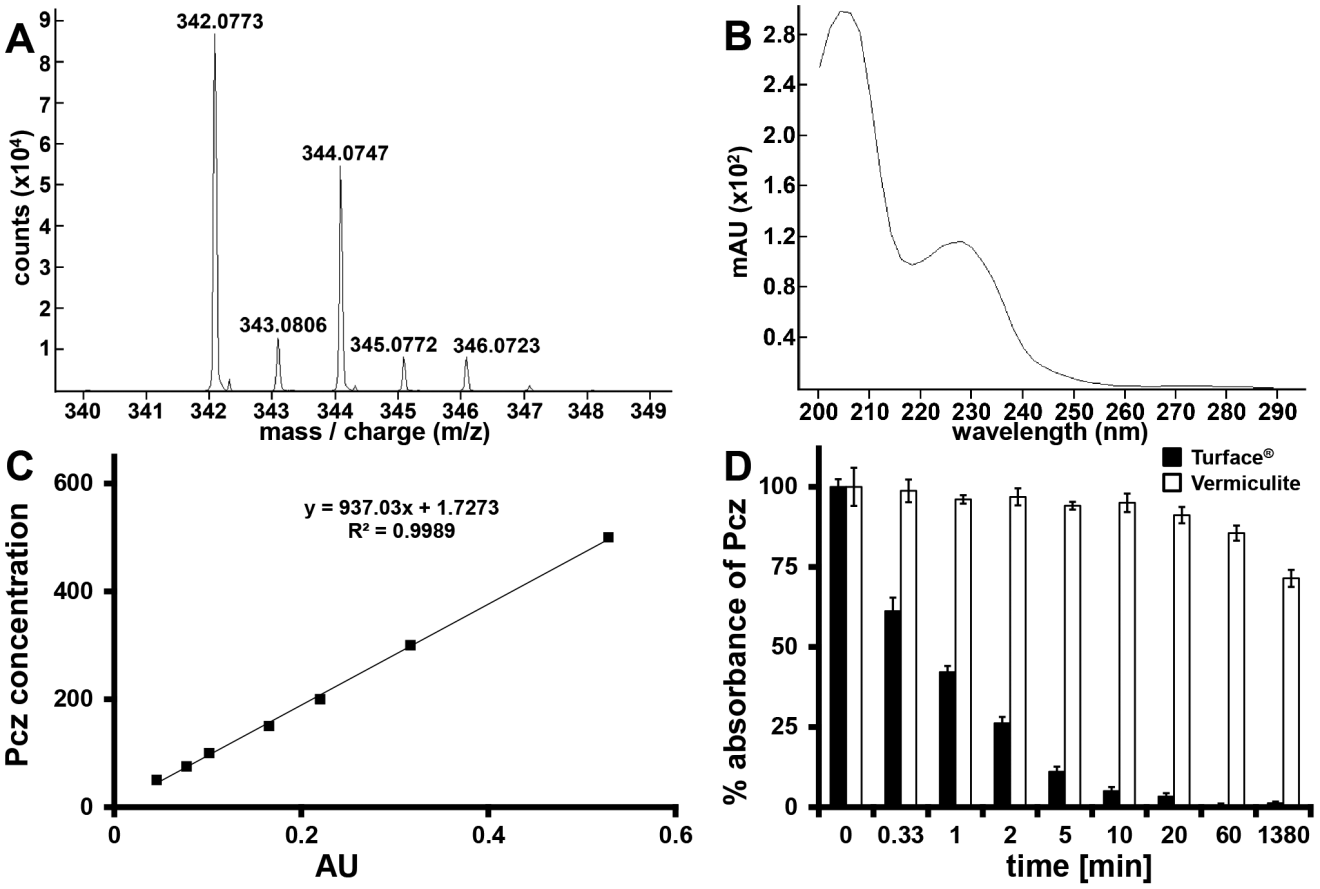
LC-MS, and standard curve of Pcz within solution. (A) LC-MS spectrum of Pcz. (B) photometrically determined absorption spectrum of Pcz measured from 200 nm to 350 nm. (C) Standard curve of Pcz concentrations from 0 to 500 mM plotted against absorption at 225 nm. (D) Absorbance kinetics of Pcz on Turface (black bars) and vermiculite (white bars) measured at 225 nm. Absorbance of Pcz to substrates was tested from 0 to 23 h.

Although these results demonstrated the effect of growth substrates on Pcz potency, the question of how Pcz action is affected remained open. We analyzed the spectral absorbance of Pcz combined with high-pressure liquid chromatography-mass spectrometry (HPLC-MS) to confirm the identity and quantify the presence of Pcz in solution. Pcz was identified in the HPLC based on its exact mass and natural isotope distribution, with an overall confidence score of 98.3% (. 4 A). The correct empirical formula was predicted, with an exact mass error of −1.2 ppm. Two local UV maxima in the absorption spectrum of Pcz at 204 and 225 nm were detected (Fig. 4 B). Quantification of Pcz was based on the absorption at 225 nm in order to reduced background interference. The local Pcz maximum at 225 nm showed a linear correlation (R^2^ = 0.9989) with a series of spiked concentrations of Pcz in solution (Fig. 4 C). The Pcz absorbance spectrum allowed us to comparatively analyze the interaction kinetics of Pcz with media substrates. We chose the two clay-derived substrates vermiculite and Turface as they represent the two extremes in terms of Pcz efficacy and are both suitable substrates for maize growth under greenhouse conditions (Fig. 4D). A one-hour exposure of 500 µM Pcz to equal volumes of Turface and vermiculite reduced free Pcz in the supernatant by 99.7% and 14.5%, respectively. Even at equal weight, we detected only 58.2% of Pcz remaining in the supernatant after one hour of exposure to 2 g of Turface. This significant difference in Pcz depletion from the supernatant was more pronounced after an exposure of 23 h to vermiculite and Turface. When 500 µM Pcz was exposed to 2 or 20 g of Turface for 23 h, only 5.7% or 1.2% Pcz remained in the supernatant, respectively (S1 Figure). In contrast, 71.4% of Pcz was detected in the supernatant after 23 h exposure to vermiculite (Fig. 4 D). These results indicate that while the free Pcz concentration rapidly decreased in solutions exposed to Turface, it mostly remained in solution when exposed to vermiculite.

### Pcz binds with high affinity to Turface media

Our results suggested that Pcz is depleted from the supernatant after contact with Turface and to a lesser extent vermiculite, but the actual cause remained elusive. Given that the cation exchange capacity (CEC) of vermiculite (100–150 meq/100 g) [47] is actually higher than that of Turface (33.6 meq/100 g) (personal communication Profile Products LLC) electrostatic interactions are unlikely the driving force of Pcz/Turface interactions. Either the surface composition and/or the hydrophobic interactions of Turface could be responsible for its strong binding to Pcz. As a first step to test this hypothesis, we determined the chemical composition of Turface. Our data indicates that Turface primarily consists of kaolinite, illite, and quartz, with a lack of organic components (S2 Figure). Next, we used Fourier Transform Infrared Spectroscopy (FTIR) to determine whether Pcz directly binds to the surface of calcined clays such as Turface.

**Figure 5:**
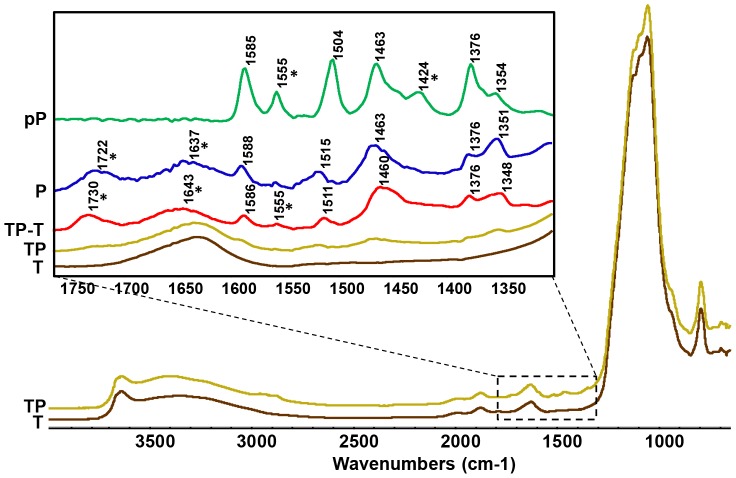
FTIR. Diffuse Reflectance (DR- FTIR) spectra of (T) Turface and (TP) Turface saturated with Pcz are shown in the 4000 to 700 cm^−1^ region. An expanded plot showing the spectra features of interest are plotted in the inset from 1750 to 1350 cm-1 showing the DR-FTIR spectra of (T), (TP), and (TP-T) the spectral subtraction of (TP) – (T). For comparison, the Attenuated Total Reflectance (ATR-FTIR) spectrum of (P) Pcz and (pP) pure Pcz are shown. The bands marked by a (*) are spectral features influenced by the presence of the surfactant in (P) Pcz.

FTIR spectra of Turface, Pcz, and Pcz sorbed to Turface were recorded (Fig. 5). The FTIR spectrum of Turface has features that match spectra of the Si-O stretch (1000–1050 cm^−1^), the HOH bending region (1630 cm^−1^), and sorbed water (3000–3600 cm^−1^) (Fig. 5, brown T-line). Included in Fig. 5 is the spectrum of pure Pcz (Fig. 5, blue P-line).

The spectrum of Pcz sorbed to Turface showed the same features of the Si-O stretch, HOH bending region and sorbed water, however, with additional bands in the range from 1750 to 1350 cm^-1^ (Fig. 5, yellow TP-line). The spectral subtraction of the Turface spectrum from the spectrum of Pcz sorbed to Turface shows that the majority of the bands between 1750 and 1350 cm^−1^ match the absorbance spectrum of Pcz in this range (Fig. 5, red TP-T-line). The Pcz-specific spectral features are clearly observable on the spectra of Turface and Pcz mixtures with discernible features at 1348 cm^−1^, 1376 cm^−1^, 1460 cm^−1^, 1511 cm^−1^, and 1586 cm^−1^ (Fig. 5). The formulation of Pcz used in the experiments of Pcz sorbed to Turface and in the root drench experiments contains surfactants and excipients to better solubilize Pcz. To ensure that the observed bands identified on the Pcz sorbed to Turface spectrum in fact correspond to Pcz and not surfactants, we also tested pure Pcz with FTIR (Fig. 5, green pP-line). The spectral characteristics of pure Pcz are consistent with literature values reported for Pcz and related triazoles. [50], [51] The results suggest that the spectral band at 1730 cm^−1^ observed in the TP-T-line (red) and P-line (purple) (Fig. 5) is due to the 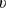
(CO) or the surfactant. Additional discernible surfactant bands (*) are found at 1424 cm^−1^ and 1555 cm^−1^ (pP), 1637 cm^−1^ and 1722 cm^−1^ (P), 1555 cm^−1^, 1643 cm^−1^, and 1730 cm^−1^ (TP-T).

### Adsorption isotherms of Pcz with vermiculite and Turface

**Figure 6:**
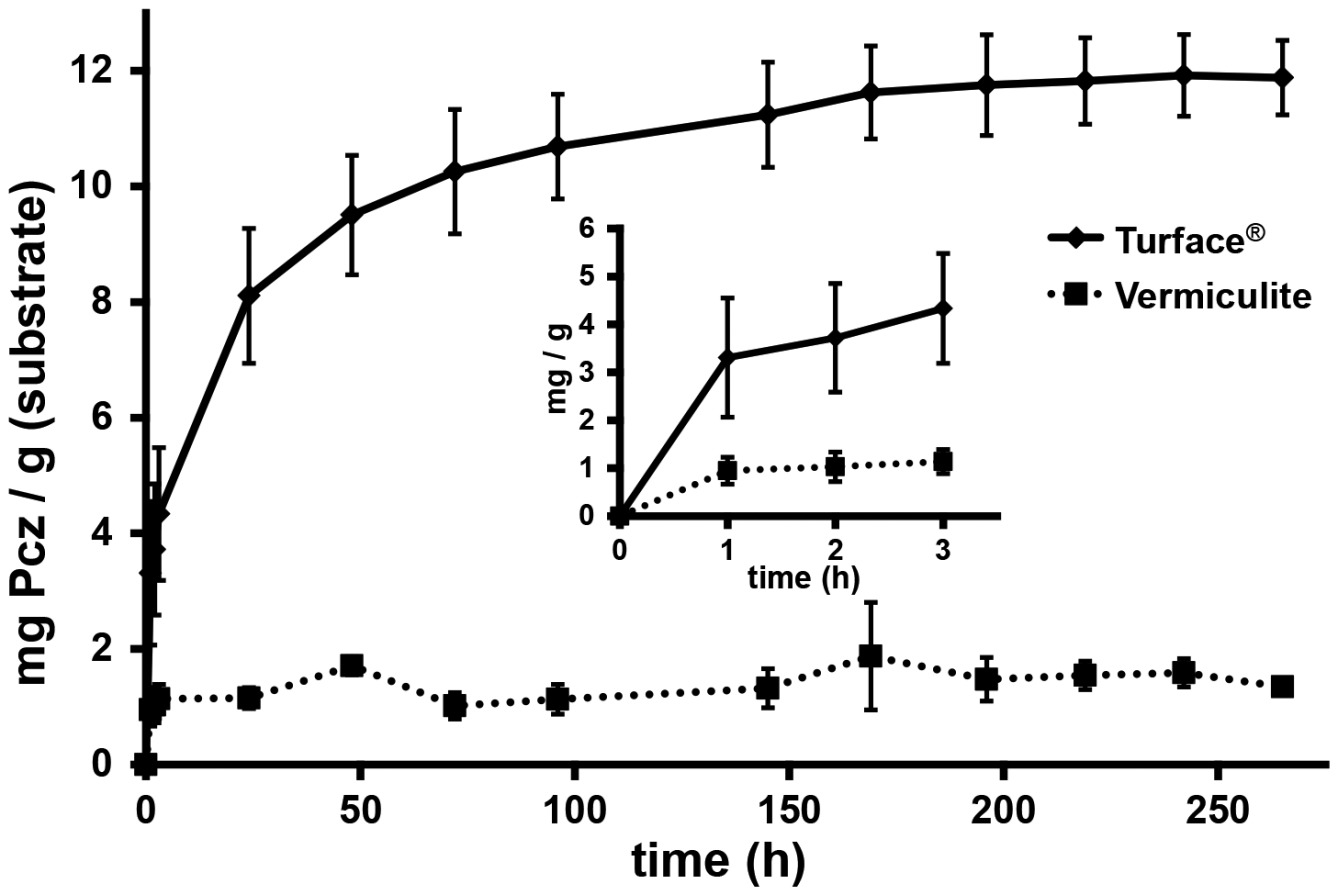
Adsorption isotherms of Pcz to Turface and vermiculite. Adsoprtion isotherms of Pcz absorbance to Turface and vermiculite determined over 250 h. Inset shows absorbance kinetics during the first 3 h of interaction. Error bars represent standard deviation.

**Figure 7:**
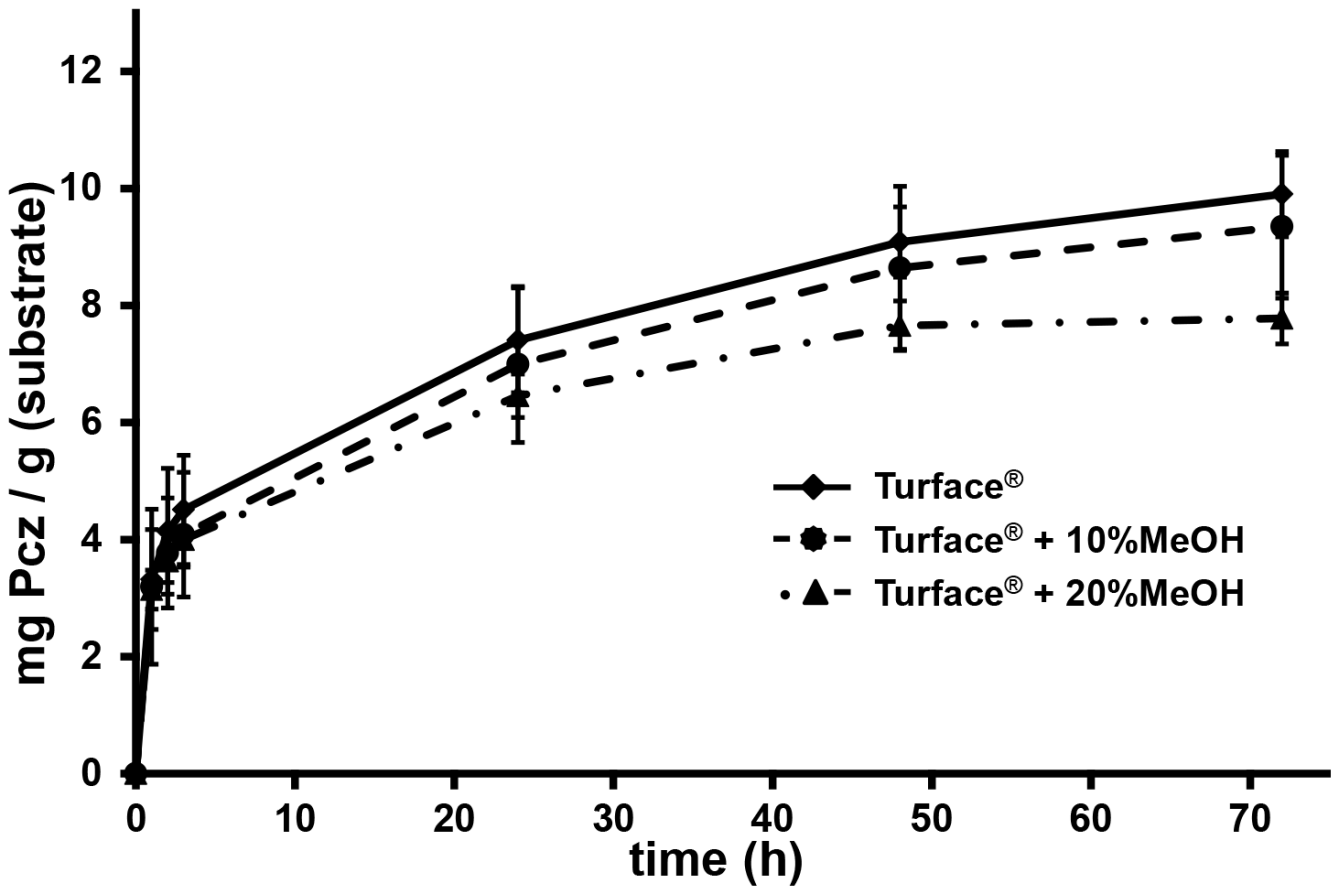
Methanol inhibits Pcz adsorption to Turface. Adsorption isotherms of Pcz absorbance to Turface over 72 h with Pcz in water, Pcz in 10% MeOH, and Pcz in 20% MeOH. Error bars represent standard deviation.

To further test the hypothesis that Pcz directly binds to Turface and to measure the binding capacity of Pcz to Turface we determined its adsorption isotherm by saturating Turface with Pcz. We found that 3.3 mg Pcz was adsorbed by 1 g Turface in the first hour, and that the binding equilibrium was not reached until 242 h of incubation with a total of 11.9 mg Pcz/g Turface adsorbed. Conversely, vermiculite showed a relatively low capacity to bind Pcz. Binding equilibrium was reached at a total of 1.33 mg Pcz/g vermiculite, and remained stable over the 265 h time course (Fig. 6). Not only was vermiculite's binding capacity to Pcz nine fold lower, but saturation was reached after about 1 h of exposure with 0.95 mg Pcz/g vermiculite adsorped (Fig. 6). To test the hypothesis that hydrophobic interactions are the cause for Pcz adsorption to Turface we determined the rate of absorbance of Pcz to Turface in 10% or 20% methanol (MeOH). We observed that after 72 hours 5.5% less Pcz was adsorbed to Turface in 10% MeOH, and 11.9% less Pcz absorbance occurred in 20% MeOH, respectively, compared to the water control (Fig. 7).

### Growth media substrates show differential interactions with applied chemicals

**Figure 8:**
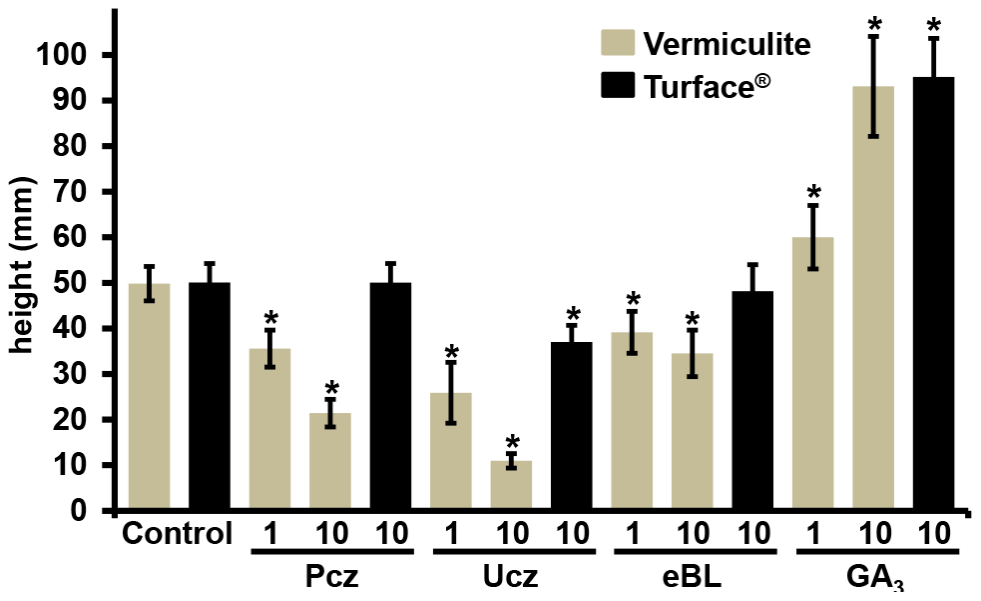
Growth responses of B73 seedlings to Pcz, Ucz, eBL and GA_3_ on Turface and vermiculite media. Light grown B73 seedlings grown on Turface (black bars) and vermiculite (grey bars) treated with 1 and 10 µM Pcz, Ucz, eBL and GA_3_ for 9 d. Seedlings grown on vermiculite were treated with 1 and 10 µM concentration of each chemical. Seedlings grown on Turface were treated with 10 µM concentrations of each chemical. Error bars represent standard deviation. Statistically significant differences to controls are indicated with asterisks determined by student's t-test (*p*<0.01).

We investigated whether the inhibitory effects of Turface and peat substrate mixes on Pcz efficacy were unique or also applied to other PGRs. In comparison to Pcz we tested the phytohormones eBL and GA_3_, as well as the growth inhibitor Ucz. B73 seedlings grown in vermiculite treated with eBL, Pcz, or Ucz at concentrations of 1 or 10 µM produced dark green leaves accompanied by a dwarfed plant stature. In particular, 10 µM eBL, Pcz, or Ucz reduced the height of seedlings by 31%, 57%, or 78%, respectively, when compared to controls (Fig. 8). For Turface grown plants neither eBL nor Pcz treatment showed a significant change in plant height compared to controls (p = 0.98, eBL and p = 0.19, Pcz). Although the efficacy of Ucz in Turface was reduced compared to vermiculite, seedlings treated with 10 µM Ucz in Turface were 26% shorter compared to controls. Interestingly, the growth promoting effect of exogenously applied GA_3_ was not significantly different (p = 0.45) in either Turface or vermiculite. A drench application of 10 µM GA_3_ increased the height of vermiculite and Turface grown plants by 87% or 90%, respectively (Fig. 8).

### Pcz efficacy under media-free culture conditions

**Figure 9:**
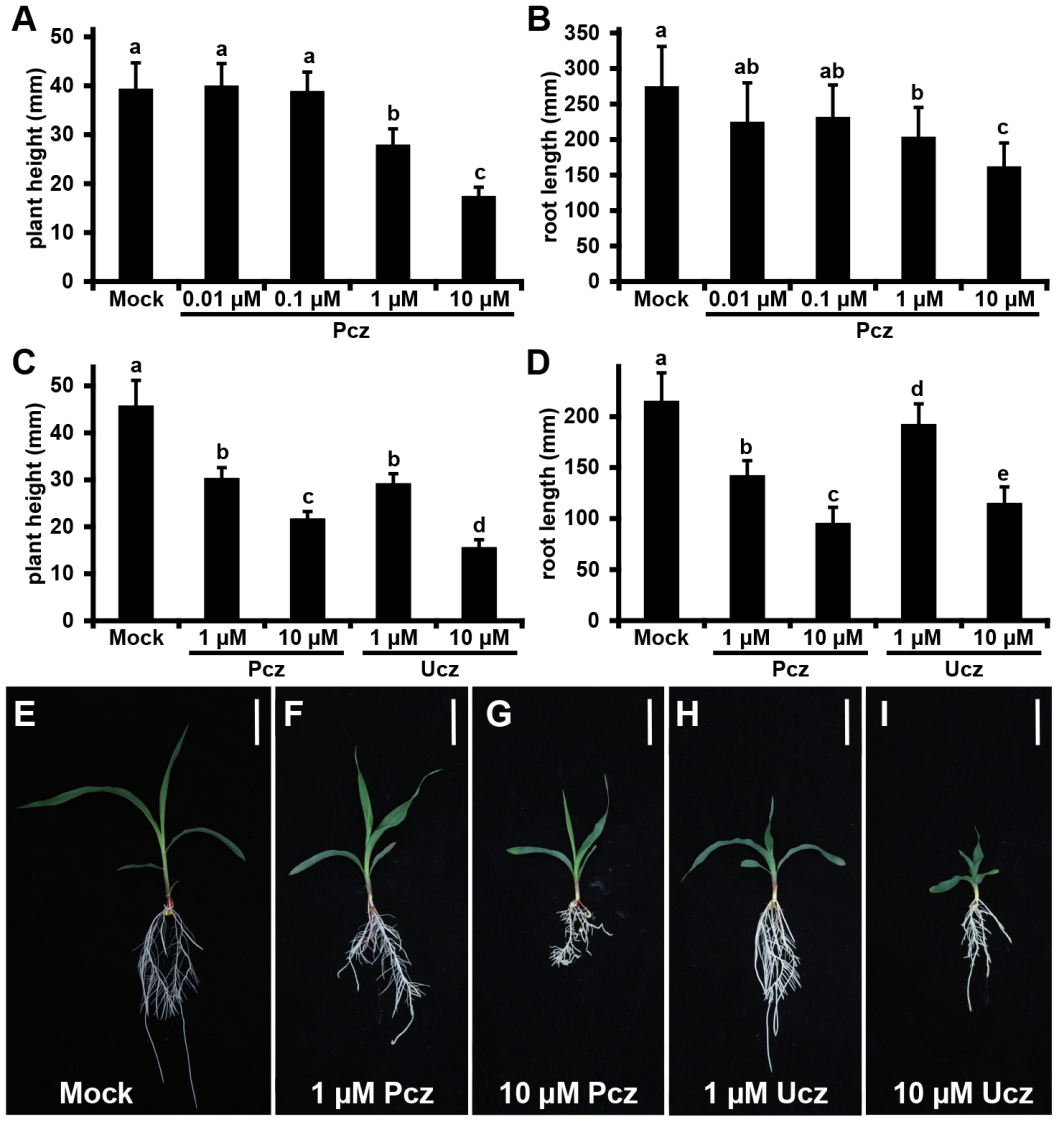
Plant growth responses of maize seedlings treated with Pcz or Ucz grown in an aeroponic culture system. B73 maize seedlings were germinated and grown as described in Material and Methods and treated with (A–B) Mock conditions, 0.01 µM Pcz, 0.1 µM Pcz, 1 µM Pcz, or 10 µM Pcz. A second set of plants was treated with (C–D) Mock, 1 µM Pcz, 10 µM Pcz, 1 µM Ucz, or 10 µM Ucz. (A,C) plant height measured from the top of the mesocotyl to the second leaf collar. (B,D) length of the primary root measured from the root tip to the root-shoot transition zone. All plants were grown in aeroponic culture (see also Fig. S3). Error bars represent standard deviation. Statistical analysis was performed by “Post Hoc” and indicated by lowercase letters (p<0.05). (E–I) Photographic images of seedlings grown in aeroponic culture. Seedlings were grown in the presence of (F) 1 µM Pcz, (G) 10 µM Pcz, (H) 1 µM Ucz, and (I) 10 µM Ucz. Scale bars indicate 5 cm.

Our previous results established vermiculite as a more suitable medium for phytohormone studies, at least for the growth regulators tested here. However, adsorption isotherm results showed that vermiculite also binds small amounts of Pcz (1.33 mg/g). This raises the question whether Pcz efficacy is still reduced due to its interaction with vermiculite. One way to investigate this question is to completely circumvent the use of media substrates through the use of aeroponic culture. Instead of using solid growth media, aeroponic systems rely on the use of mists or aerosols to deliver both nutrients and chemical treatments to the plant root system [52] (S3 Figure). In order to minimize the effect of the application methods we applied Pcz in aeroponic culture as a mist spray to the root zone only. To determine the minimum concentration necessary to significantly affect plant growth in aeroponic culture we applied Pcz ranging from 0.01 to 10 µM. A concentration of 1 µM Pcz or higher was sufficient to significantly decrease both shoot and root length (Fig. 9 A–B). The potency of Ucz was similar to Pcz; in that, concentrations of 1 µM significantly reduced plant height and root length compared to controls. Treatments with 10 µM Pcz or Ucz reduced seedling height compared to controls by 53% and 66%, respectively (Fig. 9 C, G–I). Root lengths decreased similarly by 55.7% and 46.5%, respectively (Fig. 9 D, G–I). As observed previously in plants treated in vermiculite, Pcz or Ucz applied in aeroponics induced not only dwarfism but also caused darker green leaves (Fig 9 E–I). This phenotype was also observed in BR-defective mutants in *Arabidopsis* [53]. Our results are supported by chlorophyll content index (CCI) measurements, which showed a significant increase in the CCI of at least two to three-fold for plants treated with 1 µM Pcz or Ucz (S4 Figure). Interestingly, although a significant difference in growth inhibition between 1 and 10 µM treatments was observed, the CCI appeared to remain stable at 1 µM with no further increase in treatments with higher concentrations of Pcz or Ucz (S4 Figure).

## Discussion

Growth promoting hormones, such as GA and BRs, have been a focal point of research due to their potential to increase the harvest index or biomass production of agriculturally important crops [54], [55]. The mutations and their corresponding genes that enabled the Green Revolution in wheat and rice have been identified. They relate to either gibberellin metabolism (*sd1* in rice) or signal transduction (*Rht1* and *Rht2* in wheat) [56]. But the positive effects of the Green Revolution have reached their peak as the per capita world grain production fell from 329 kg (1980's) to 313 kg (1990's) [57]. Using phytohormone biosynthesis inhibitors to increase harvest index instead of hormone deficient mutants allows them to be applied across many species without the development of extensive breeding programs. The use of chemical inhibition has the additional advantage to be used selectively (organ or developmental specific), which minimizes the effect of developmental and physiological differences between wild type and deficient mutant plants. In addition to their value for scientific research, both hormones and their respective biosynthesis inhibitors are often the active ingredients of PGRs used to improve and regulate growth and productivity in horticultural and agricultural production.

One key advantage of MS agar media as a preferred media for model species such as *Arabidopsis* is that it hardly affects biochemical treatments. However, to sustain growth of larger crop species until maturity, MS agar media is often inadequate. Very little is known about how other common growth media impact chemical treatments. In this study we focused on the quantitative impact and mechanism of media interaction with biochemical treatments. Pcz, a triazole compound, is commercially used as a fungistat against a broad range of phytopathogenic fungi. Its fungistatic mode of action is the blocking of lanosterol 14R-demethylase (CYP51A1), similar to that of Ucz [58], [59]. More recently, Pcz has been characterized as a potent and specific BR biosynthetic inhibitor with increased availability and reduced costs compared to other BR inhibitors. Consistent with previous reports from *Lepidium sativum*, we observed Pcz induced BR deficient phenotypes in treated maize B73 plants [1], [9], [13]. The severity of these phenotypes, such as dwarfism, greatly varied dependent on the growth substrate used. The greatest efficacy of Pcz was observed in both vermiculite and perlite substrates. Whereas, a 20 µM Pcz treatment in vermiculite inhibited seedling growth by more than 60%, an increase of 10-fold in Pcz concentration only decreased plant height by a further 10%. This suggests that 20 µM Pcz nearly saturates the inhibition of BR biosynthesis when seedlings are grown in these media. In contrast, Turface significantly lowered Pcz efficacy. Even a treatment with 200 µM Pcz decreased height in Turface grown seedlings by only 25%. These results support our hypothesis and illustrate the dramatic differences in the total Pcz binding capacity as well as the binding kinetics between the two different growth substrates.

Given its widespread use as a fungicide, Pcz has been studied for its potential effects on the environment [60], [61] and in mammalian systems [62], [63]. It has previously been shown that Pcz binds to a greater extent to silty clay loam soils compared to sandy loam clay soils. The rates of degradation and mineralization of Pcz have been found to be greater in sandy loam soils than in silty clay loam soils [64], [65]. An additional study showed that different size fractions of soil given their different chemical and physical properties had different capacities to bind to Pcz [66]. Although these investigations studied the long-term effects of Pcz in soil interactions, their results offer two likely hypotheses for observed differences between the two extremes Turface and vermiculite and their effects on Pcz efficacy. Pcz is either bound by soil particles and therefore disallows uptake by the plant at least in short term applications, or is degraded by the soil interaction by hydroxylation of the n-propyl side chain and the dioxolane ring, as previously reported [67].

In conjunction with previous reports we were able to detect Pcz within solution using HPLC-MS [68]. The presence of a dioxalene ring in its chemical structure made it possible to use the absorption spectrum of Pcz as a suitable alternative for detection in solution. The local absorption maximum at 225 nm correlated with the concentrations of spiked Pcz and allowed us to measure the Pcz availability in solution over 23 h of interaction with either Turface or vermiculite. We found that less than 5% of Pcz remained in the supernatant after 5 min of interaction with Turface, which explains the low efficacy of Pcz on plants grown in this medium. Conversely, more than 70% Pcz remained in the supernatant after 23 h in vermiculite. This would allow high uptake by the root system and may explain the high efficacy of Pcz on plants grown in this medium. Although both media are used at equal volume for planting, even at equal weight significantly lower amounts of Pcz remained in the supernatant of a Turface-Pcz solution compared to vermiculite-Pcz solution. Our results are supported by data from Kim *et al*. [65], which demonstrated high affinity of clay soils to Pcz.

To investigate if Pcz is directly bound by Turface we used FTIR. The spectra provide direct evidence that the Pcz is adsorbed to Turface and therefore interferes with uptake of the inhibitor by the plant. The spectral features of Pcz are minimally perturbed by Turface and do not provide clear insight into the binding mode of Pcz to Turface. Based on the low solubility of Pcz, a likely mechanism would involve hydrophobic interactions of Pcz with the calcined siloxane surface of Turface [69]. If the different binding kinetics between Turface and vermiculite would be the main reason for their different Pcz efficacy, one should have expected differences in the amount and time necessary for saturation. Adsorption isotherm of vermiculite showed near saturation after one hour and only 1.3 mg Pcz/g vermiculite was bound. In contrast, Turface was able to bind nearly 10 times more Pcz, with a maximum of about 12.5 mg Pcz/g Turface. Furthermore, saturation equilibrium of Turface with Pcz was not reached until 250 h. Together with the 10 times higher specific weight of Turface compared to vermiculite, this indicates approximately 100-fold lower Pcz availability for equal planting volumes in Turface. The long time span to saturate Turface with Pcz (10 d) could also be a contributing factor to its low Pcz potency. Although the rate of degradation of Pcz has been shown to be lower in clay type soils with a half-life of more than one year [65], different degradation of Pcz may occur in vermiculite and Turface [70]. Given that Pcz was stable even after 10 d suggests that biodegradation is not a major factor for Pcz efficacy. However, we cannot rule out that chemical modifications did not change Pcz absorption and influence its efficacy. Previous reports show that the degradation of Pcz by hydroxylation of the n-propyl side chain and the dioxolane ring, as well as with formation of 1,2,4-triazole, could affect the absorption spectrum of Pcz [65].

The differences in Pcz efficacy observed between Turface and vermiculite raised the question whether this effect was specific to Pcz or is characteristic for many PGRs. Hence we tested the efficacy of Ucz, eBL, and GA_3_ in both growth media. Similar to Pcz, high concentrations of eBL drench treatment were ineffective in Turface but showed an expected reduction in plant height when grown in vermiculite. High concentrations of eBL have a growth inhibiting effect in plants, as exogenous eBL induces a negative feedback mechanism, which down-regulates expression of BR biosynthesis genes [71]. Although Ucz efficacy was reduced in Turface -grown seedlings compared to vermiculite-grown seedlings, the Ucz activity measured in height reduction of seedling growth was greater than for Pcz or eBL in Turface (Fig. 8). Interestingly, we did not find a quantitative difference for growth effects of GA_3_ in drench applications in either, Turface or vermiculite. In both cases, 10 µM GA_3_ almost doubled the size of treated plants (Fig. 8). One possible explanation for the efficacy differences of the four tested PGRs could be their solubility in water. GA_3_ is a much more hydrophilic compound (solubility 5g/l) compared to Pcz (100 mg/l), Ucz (8.41 mg/l), or eBL (5 mg/l). Pcz has a pKa of 1.09, which would generate its protonated form only at very acidic conditions not present in the tested environment. Therefore, Pcz is mostly neutral in solution, which makes it highly hydrophobic. Turface is made up of kaolinite, illite, and quartz calcined at 650°C (personal communication Profile Products LLC). The hydrophobic nature of kaolinite and illite is further enhanced by calcination and thus creates more intense hydrophobic areas on the siloxane surface of Turface [72]. This could be an explanation for strong physical interaction observed between Pcz and Turface. In order to explore the proposed van der Waals binding of Pcz to Turface, a sorption experiment of Pcz to Turface was conducted with varying amounts of MeOH. MeOH has a reduced polarity index of 5.1, whereas water has a polarity index of 9.0. Both solvents are completely mixable, which makes MeOH an ideal solvent to study hydrophobicity effects in aqueous solvent mixes [73], [74]. MeOH is commonly used as a co-solvent in sorption studies of non-polar organic compounds [75]. The amount of Pcz sorbed to Turface was systematically lower with increasing concentration of MeOH. The addition of MeOH makes the solvent less polar and reduces the sorption and affinity of Pcz for the hydrophobic surface of Turface.

Vermiculite is high-density charged clay and therefore strongly hydrophilic, which reduces interactions with hydrophobic compounds such as Pcz. This hypothesis is supported by lack of interaction observed for charged GA_3_ with Turface and given the similar negative charge of GA_3_ and vermiculite, this may also explain the lack of interaction between them. Together our data indicates that Pcz, Ucz, and eBL interacted with Turface through hydrophic interactions but not as readily with the highly charged vermiculite. Although we cannot rule out an influence of surfactants in the case of Pcz and Ucz, both eBL and GA_3_ were supplied in ethanol without additional surfactants.

Although the Pcz binding capacity of vermiculite was significantly lower than that of Turface, the question remained whether vermiculite negatively impacts Pcz effectiveness. We elucidated this by testing Pcz applications without media substrates. Concentrations of 1 µM Pcz or higher significantly reduced both plant height and root growth compared to mock treatments (Fig. 9). This suggests either active transport of Pcz to the shoot zone, or that an inhibition of BR biosynthesis in the root is sufficient to inhibit shoot growth. Comparative analyses showed that 1 µM is also the threshold for Ucz to significantly inhibit shoot and root growth (Fig. 9 C-D). In addition to the effect on shoot and root length both Pcz and Ucz induced morphological changes. Pcz showed phenotypes reminiscent of BR deficient plants with reduced leaf sheath elongation, twisting, upright and dark green leaves [9]. Ucz also induced a dwarf stature with wide dark green leaves similar to maize plants deficient in GA biosynthesis [76]. The leaf CCI of plants treated with 1 µM Pcz or Ucz was nearly double compared to mock, whereas 10 µM of Pcz or Ucz showed no significant further increase in CCI. Dark green leaves are a classical phenotype of both BR and GA deficient mutants. In the case of Pcz or Ucz the increase in CCI is likely due to an increase in chlorophyll production [77], reduction in cell size as observed in *Arabidopsis* [53], or a combination of both [78].

## Conclusion

The choice of media substrate is rarely considered an important aspect of the experimental design and is primarily based on optimizing the growth conditions for the plant. However, our results illustrate how drastically the tested media substrates influence the efficacy of various chemical growth regulators. The ability of calcined clay substrates to impair the efficacy of triazole-based PGRs is based on their hydrophobic interactions. This emphasizes the importance of appropriate media selection to balance growth conditions with the effectiveness of biochemical treatment studies. Vermiculite is an applicable medium for chemical treatment bioassays, as it minimally interacts with applied compounds. Turface is a more suitable medium for long-term growth of larger plants, such as maize, however it strongly inhibits the efficacy of hydrophobic soil-drenched PGRs.

## Supporting Information

S1 Figure **Absorbance kinetics of Pcz on Turface.** Absorbance values of Pcz in the supernatant at 225 nm when interacting with 2 g (grey bars) or 20 g (black bars) of Turface tested from 0 to 23 h. Error bars represent standard deviation.
(TIF)

S2 Figure **X-ray diffraction of Turface.** X-ray diffraction pattern of Turface. Analysis was obtained using a PANalytical X-ray diffractometer (model X′Pert PRO; Natick, MA) using Co radiation. Software analysis identified that its main components are kaolinite (K), illite (I), and quartz (Q). Major characteristic peaks are indicated.
(TIF)

S3 Figure **Aeroponic System.** (A) Overview of aeroponics setup in a greenhouse with 24 spray containers in groups of three, bearing up to eight plants each. (B) 20 l steel pressurized tanks with maintained pressure by air compressor. Hoses for input of pressurized air and output into the spray buckets are connected to the tanks. Each tank feeds one group of three spray buckets. (C) Bottom of a 20 l spray bucket with microjet nozzle to spray the root space and run-off outlet to remove excess spray solution. (D) Air conditioning unit to control temperature of the root zone and Chrontrol XT series electronic timer device to control frequency and duration of spray treatment. (E) Maize seedlings mounted into styrofoam tops after two weeks of aeroponics culture.
(TIF)

S4 Figure **Chlorophyll content measurements (CCI) of Pcz and Ucz treated seedlings.** Chlorophyll content index of B73 seedlings grown in silica sand for 6 d then transplanted to aeroponic culture for 9 d when measurements were taken. CCI was measured directly in the middle of the second leaf between the leaf collar and leaf tip using a CCM-200 chlorophyll content meter (Opti-Sciences, Hudson, NH). Error bars represent standard deviation and lower class letters indicate significant differences between treatments as determined by “Post-hoc” test (*p*<0.05). *n* = 12.
(TIF)
