# Supplementary figures and images for: Soilless Plant Growth Media Influence the Efficacy of Phytohormones and Phytohormone Inhibitors
Source: PLoS One. 2014 Dec 8;9(12):e107689. doi: 10.1371/journal.pone.0107689 (PMC4259294; doi:10.1371/journal.pone.0107689)

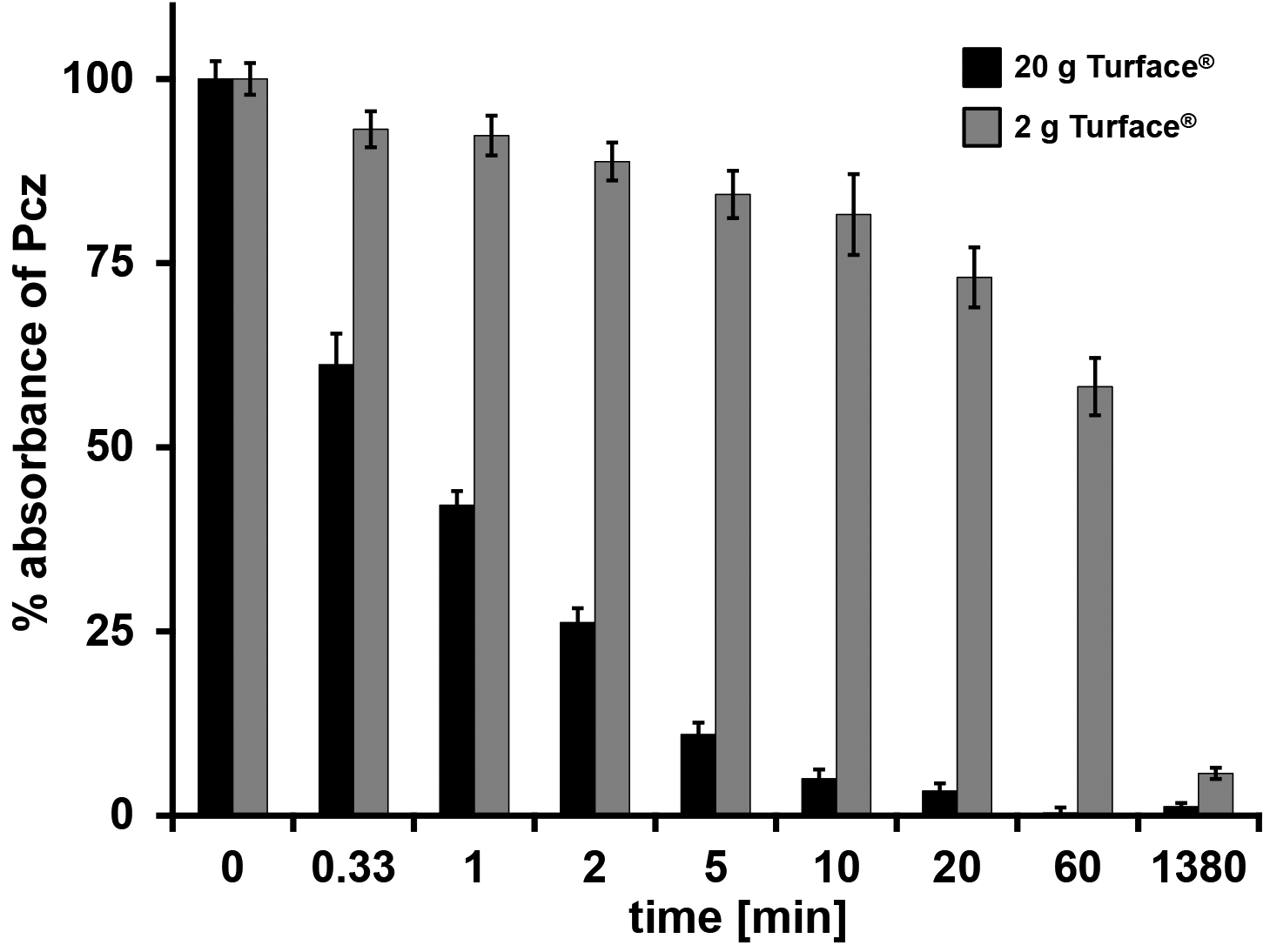

Supplement: S1 Figure — Absorbance kinetics of Pcz on Turface. Absorbance values of Pcz in the supernatant at 225 nm when interacting with 2 g (grey bars) or 20 g (black bars) of Turface tested from 0 to 23 h. Error bars represent standard deviation. (TIF) [file pone.0107689.s001.tif]

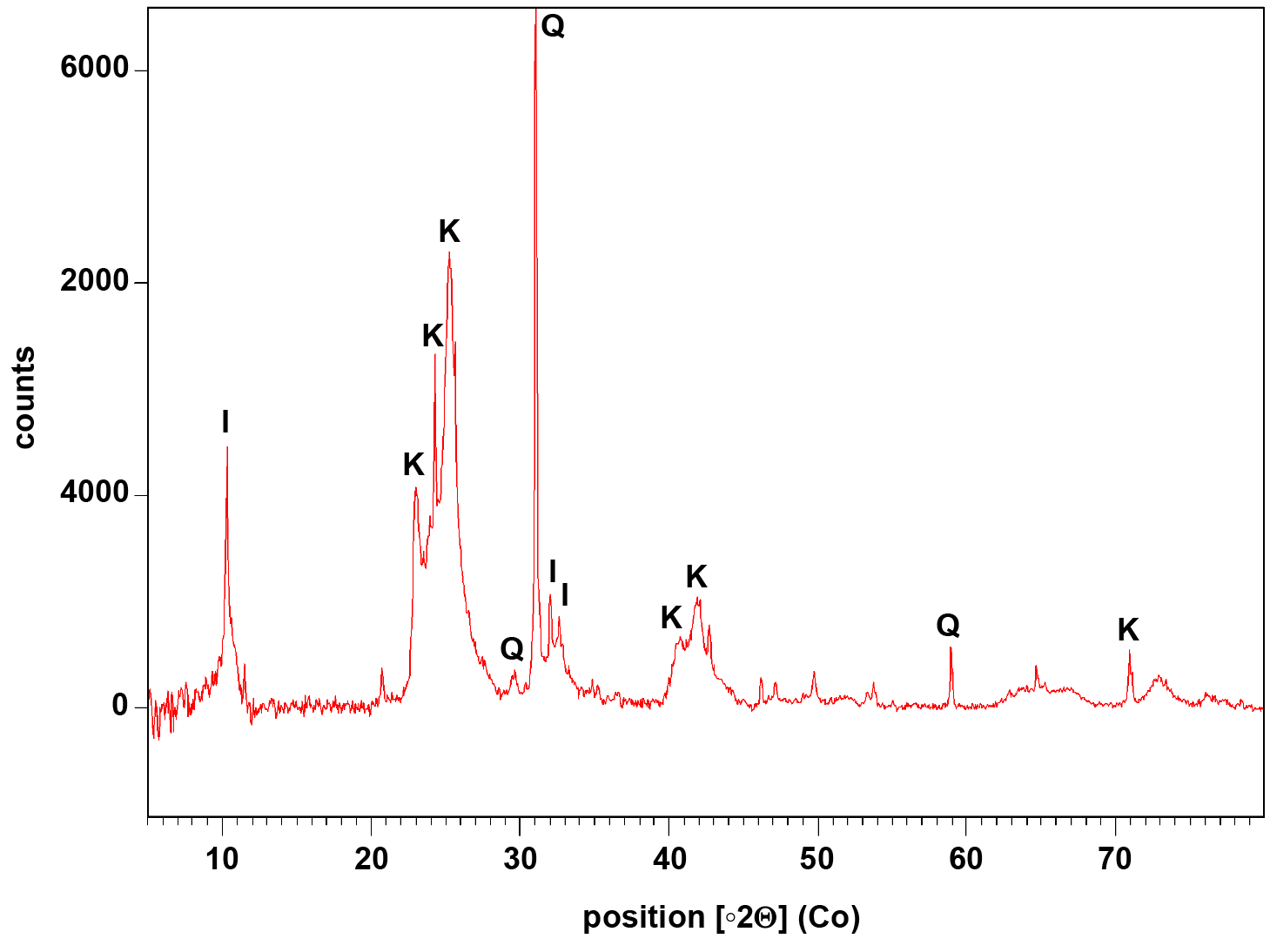

Supplement: S2 Figure — X-ray diffraction of Turface. X-ray diffraction pattern of Turface. Analysis was obtained using a PANalytical X-ray diffractometer (model X′Pert PRO; Natick, MA) using Co radiation. Software analysis identified that its main components are kaolinite (K), illite (I), and quartz (Q). Major characteristic peaks are indicated. (TIF) [file pone.0107689.s002.tif]

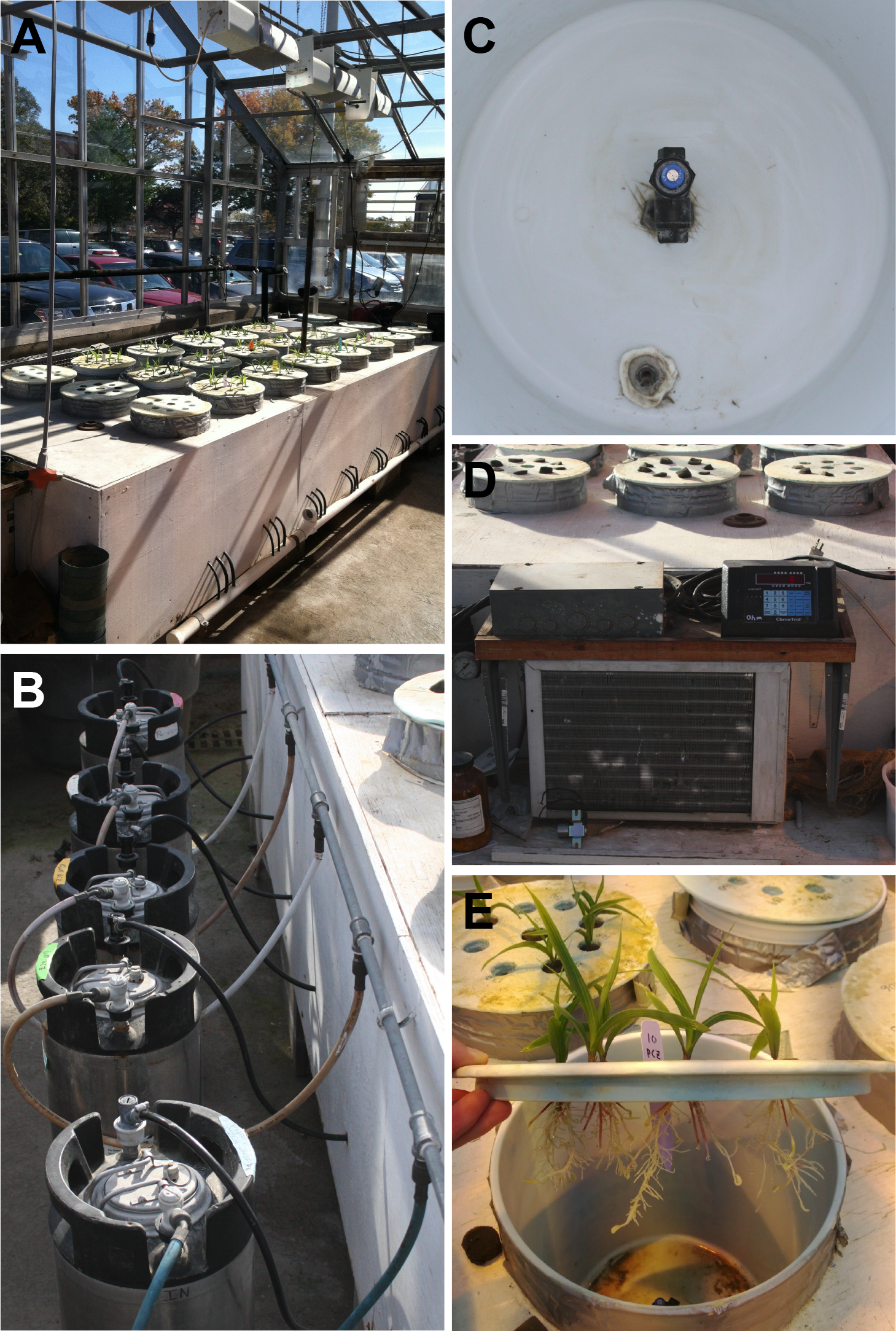

Supplement: S3 Figure — Aeroponic System. (A) Overview of aeroponics setup in a greenhouse with 24 spray containers in groups of three, bearing up to eight plants each. (B) 20 l steel pressurized tanks with maintained pressure by air compressor. Hoses for input of pressurized air and output into the spray buckets are connected to the tanks. Each tank feeds one group of three spray buckets. (C) Bottom of a 20 l spray bucket with microjet nozzle to spray the root space and run-off outlet to remove excess spray solution. (D) Air conditioning unit to control temperature of the root zone and Chrontrol XT series electronic timer device to control frequency and duration of spray treatment. (E) Maize seedlings mounted into styrofoam tops after two weeks of aeroponics culture. (TIF) [file pone.0107689.s003.tif]

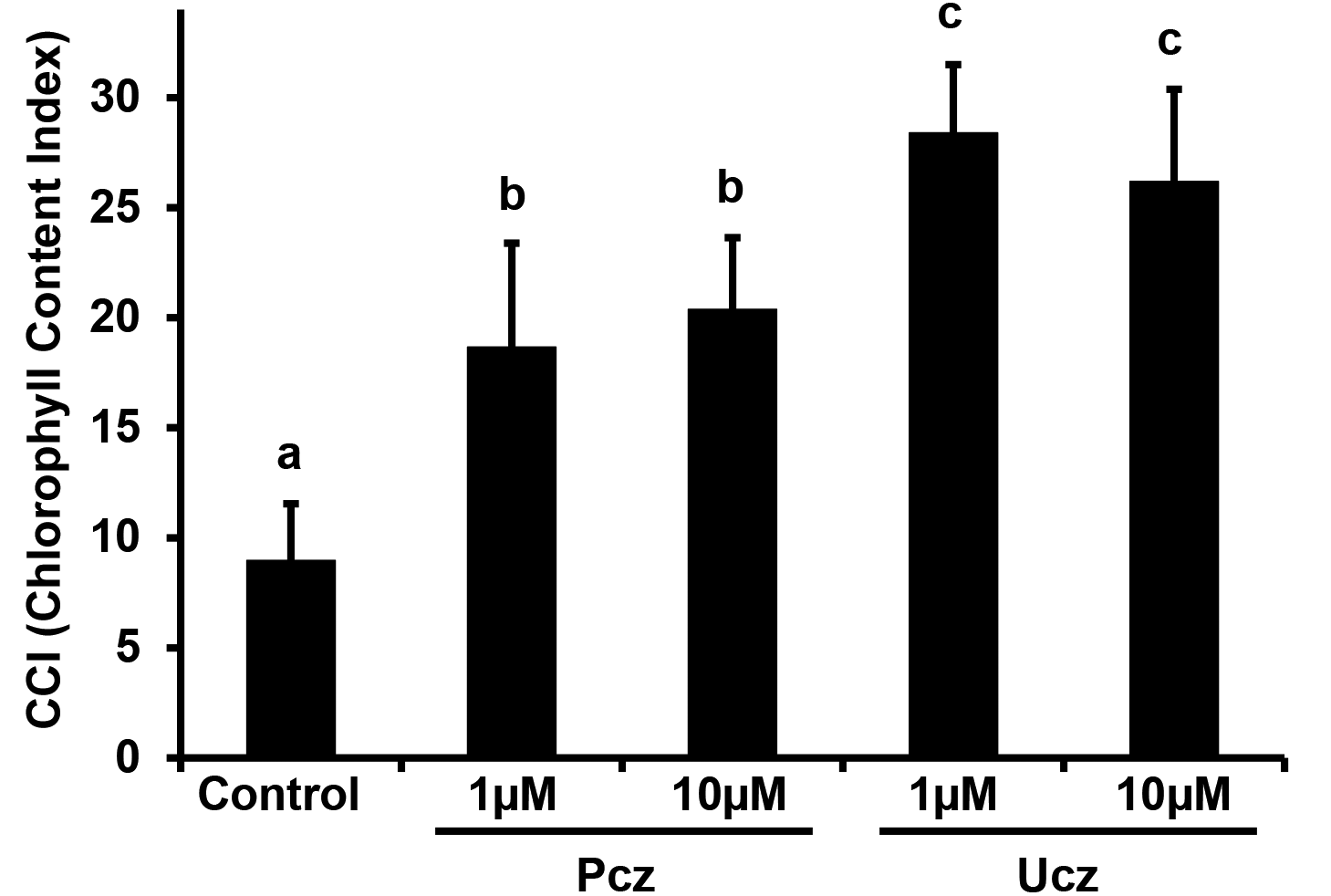

Supplement: S4 Figure — Chlorophyll content measurements (CCI) of Pcz and Ucz treated seedlings. Chlorophyll content index of B73 seedlings grown in silica sand for 6 d then transplanted to aeroponic culture for 9 d when measurements were taken. CCI was measured directly in the middle of the second leaf between the leaf collar and leaf tip using a CCM-200 chlorophyll content meter (Opti-Sciences, Hudson, NH). Error bars represent standard deviation and lower class letters indicate significant differences between treatments as determined by “Post-hoc” test (p<0.05). n = 12. (TIF) [file pone.0107689.s004.tif]
